# Determination of Multiclass Pharmaceutical Residues in Milk Using Modified QuEChERS and Liquid-Chromatography-Hybrid Linear Ion Trap/Orbitrap Mass Spectrometry: Comparison of Clean-Up Approaches and Validation Studies

**DOI:** 10.3390/molecules28166130

**Published:** 2023-08-18

**Authors:** Ourania Koloka, Marioanna Koulama, Dimitra Hela, Triantafyllos Albanis, Ioannis Konstantinou

**Affiliations:** 1 Department of Chemistry, University of Ioannina, 45110 Ioannina, Greece; raniakoloka@gmail.com (O.K.); marioanna.koulama@yahoo.com (M.K.); talbanis@uoi.gr (T.A.); 2 University Research and Innovation Center, Institute of Environment and Sustainable Development, University of Ioannina, 45110 Ioannina, Greece

**Keywords:** pharmaceutical drug, milk, QuEChERS, liquid chromatography–LTQ Orbitrap mass spectrometry, uncertainty

## Abstract

A multi-residue method was developed to identify and quantify pharmaceutical drug residues in full-fat milk, using a modified QuEChERS extraction procedure and sonication combined with Ultra-High-Performance Liquid Chromatography–High-Resolution Orbitrap Mass Spectrometry (UHPLC-HR-Orbitrap-MS). Sample preparation involves three different QuEChERS extraction procedures and sorbents for the purification step. The optimized modified extraction method, combined with the clean-up approaches using C18 and the EMR-Lipid sorbent, has been validated in terms of linearity, recovery, precision, LOD and LOQ, matrix effects (ME) and expanded uncertainty. The optimized method showed a linearity >0.9903, recoveries within the range 65.1–120.1%, precision (expressed as %RSD) <17.5%, medium (<39.9%) to low (<16.7%) matrix effects and acceptable expanded uncertainty (<33.1%). Finally, the proposed method was applied to representative real samples of milk (by local markets), revealing the existence of one pharmaceutical drug (imidocarb) in one sample.

## 1. Introduction

Pharmaceutical drugs are widely used in livestock production either for disease treatment, prophylaxis, or growth enhancement [1]. The incorrect use of these chemical compounds or improper withdrawal time may lead to the presence of residues in milk, and the consumption of contaminated milk or dairy products may expose humans to health hazards [2]. The European Union (EU) has set maximum residue limits (MRLs) for active pharmaceutical substances allowed in products of animals intended for human consumption to limit the risks related to the consumption of food containing residues of pharmaceutical drugs [3]. In addition, pharmaceuticals for human use can also be detected in food matrices as a result of their spread in environmental matrices mainly due to the disposal of biosolids in soils, the irrigation of recycled water, or from surface waters receiving wastewater treatment plant effluents. The detection of pharmaceutical drug residues in milk has been reported in previous studies [4,5,6,7,8]. Therefore, the study of pharmaceutical drug residues in food commodities, as well as the monitoring of compliance with MRLs, requires sensitive and rapid analytical methods capable of determining trace levels of a large number of pharmaceuticals in milk in a single analytical procedure.

Milk is a complex matrix containing various components, such as fat, proteins, and other components that can interfere with or prevent proper pharmaceutical drug analysis. Analytical methods for the determination of pharmaceutical drugs in milk have undergone considerable development in recent years. Milk sample preparation techniques based on liquid–liquid extraction (LLE) [9] have been replaced by other approaches, such as solid-phase extraction (SPE) [10,11], solid–liquid extraction (SLE) [5], matrix solid phase dispersion (MSPD) extraction [12,13], solid-phase microextraction (SPME) [14], and more recently by QuEChERS extraction [7,8,15,16,17,18,19].

QuEChERS is a quick, easy, cheap, effective, robust, and safe extraction compared to the above methods, which may present some difficulties related to time consumption and labor intensity, the low recovery of some target compounds, poor reproducibility, and method improvement [20]. Various modifications of QuEChERS extraction have been reported in the literature for the analysis of pharmaceutical drug residues in milk, using different sorbents for the clean-up step or a combination of them, e.g., octadecylsilane, (C18) as the usual sorbent to improve extraction on high-fat food matrices, primary secondary amine (PSA), and sodium acetate (NaOAc) [6,16,21,22]. Moreover, the Enhanced Matrix Removal-Lipid (EMR-Lipid) is a new product used as a dispersive SPE (dSPE), promising the highly selective removal of lipids, without the retention of the analyte. The EMR-Lipid sorbent was initially proposed for the analysis of pesticides in fatty matrices of plant origin [23]. It has subsequently been evaluated for the determination of several target analytes in other fatty matrices, such as bovine tissues (kidney, liver, and muscle) [24], milk [25], kale, salmon, avocado, and pork [26].

Regarding sonication, it is a technique that can effectively reduce the size of milk fat globules (MFG) via shearing, pressure fluctuations, and turbulence [27,28]. The vibrations generated by sonication can improve the extraction efficiency of the target compounds. The combination of extraction techniques can improve analyte recovery and can be efficiently applied to complex matrices such as milk. The application of the QuEChERS method in combination with ultrasound-assisted extraction for the analysis of pharmaceutical drugs in milk has been reported in some studies [29,30].

Until now, various analytical techniques have been used for the analysis of pharmaceutical drug residues in milk, mainly based on liquid chromatography (LC) systems combined with low-resolution (LR) mass spectrometry, such as LC ESI-MS or triple quadrupole LC-MS/MS (QqQ) [21,25,31,32,33,34]. The low-resolution (RL)-MS instruments mentioned above have several limitations, including a lower number of compounds that can be monitored in a single analysis, the limited ability to screen for unknown compounds, and a dependence on reference standards. LRMS requires a targeted approach involving a priori knowledge of the fragmentation pattern of the analytes (MS-MS product transitions along with optimized collision energies). On the other hand, high-resolution (HR) MS offers unique advantages, including the ability to screen samples with little or no knowledge of what is present and to create a digital data archive suitable for retrospective analysis. An LRMS measurement provides information on the nominal mass of the analyte, whereas HRMS measures the exact mass; i.e., the *m*/*z* for each ion is measured to four to six decimal places. Interferences can be discarded on the basis of accurate mass in HRMS instruments, and sample preparation procedures can be simplified, resulting in faster analytical methods. The high selectivity and mass accuracy of HRMS instrumentation can overcome false positives and false negatives when screening complex food samples [35,36].

In the last few years, high-resolution acquisition techniques have gained momentum, providing accurate mass measurements for both precursor and product ions. Liquid chromatography (LC)–high-resolution Orbitrap MS instruments offer a range of benefits for analytical applications. The use of the Orbitrap mass analyzer provides high-resolution, high-mass-accuracy, and high-quality MS/MS fragmentation, allowing the determination of an unlimited number of analytes in a single analytical run, even in complex matrices. A variety of screening methodologies can be used for residue analysis in food by LC-HRMS based on different objectives including targeted, suspected, and non-targeted or retrospective analysis. A non-targeted LC–HRMS approach for the analysis of unknown or unexpected sulfonamide residues in honey samples was investigated and optimized by Kırkan et al. [37], whereas an integrated nano LC-HR Orbitrap MS system employing a multiwalled carbon nanotube (MWCNT)-based monolithic stationary phase was applied for the analysis of antibiotics and pesticides in milk and honey by Aydogan et al. [38]. A full MS/all-ion fragmentation acquisition mode was applied in both cases. In addition, Decheng et al. [19] referred to the development of a QuEChERS extraction method for the target analysis of eight carbapenems in milk using LC–Q Exactive (QE) Orbitrap mass spectrometry. The analysis was performed in positive mode in a heated electrospray interface (HEI+) with parallel reaction monitoring (PRM).

Accordingly, the target of the present study was to establish a sensitive and rapid analytical method for the determination of multiclass pharmaceutical drugs in milk using a modified QuEChERS procedure and sonication, followed by liquid chromatography coupled to a high-resolution Orbitrap MS instrument. Different sorbents were used as dispersive SPE (dSPE) agents for the clean-up step, and the modified QuEChERS method (“AOAC 2007.01”) [39] was evaluated and fully validated. To the best of our knowledge, the optimization of the QuEChERS method combined with LC-HR Orbitrap MS in a fully validated study has not been reported so far. Finally, the optimized method was applied to 10 milk samples, which were commercially available in Greek markets for the monitoring of pharmaceutical drug residues.

## 2. Results and Discussion

### 2.1. Optimization of QuEChERS Procedure

**Figure 1:**
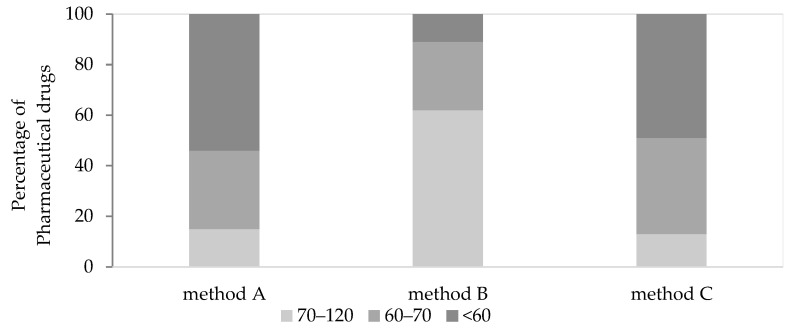
Percentages of pharmaceutical drugs exhibiting recoveries within different ranges (70–120%, 60–70%, and <60%) using different QuEChERS methods. Method A (“Original”), method B (“AOAC 2007.01”) [39], and method C (“Buffered CEN 15662”) [40].

Firstly, three primary QuEChERS extraction procedures were tested. The acetate method (method B) yielded more target compounds with recoveries ranging from 70 to 120% compared to the buffered (method C) and the original method (method A), as shown in Figure 1. More specifically, the acetate method revealed two compounds with recoveries <60%, the recoveries of five compounds ranged between 60 and 70%, and the rest (eleven compounds) exhibited recoveries from 70 to 120% (Figure 1). The next step was to operate some modifications of the acetate method to achieve higher analyte extractions together with the lower extraction of undesirable interfering compounds.

Acetonitrile was used as the extraction solvent since it is the most widely used organic solvent in the QuEChERS method. The addition of a chelating agent—EDTA 0.1 M—improves the extraction recovery of some pharmaceutical drugs by preventing their rapid chelation with metal ions [41,42]. Furthermore, the addition of EDTA could improve the adsorption of casein onto milk fat globules (MFG). EDTA is a calcium-chelating agent that can dissociate micellar calcium phosphates, resulting in a partial disintegration of casein micelles [43]. Another important parameter of the QuEChERS extraction procedure is the acidity of the extractant; therefore, the volume of formic acid in acetonitrile was tested by adding 1%, 2%, and 3.35% in the solvent. The best results were obtained after the addition of 3.35% in acetonitrile. According to Zhou et al. [16], the addition of 3.35% formic acid in acetonitrile along with the addition of sodium acetate enhances the salting-out effect and buffers the extract. Next, the contents were placed in a sonication bath (37 kHz, 100 W) for 20 min. The sonication promotes the homogenization of milk and reduces the size of the milk fat globules (MFG) [44].

Another important stage of the QuEChERS procedure concerns the selection of drying salts, which can cause phase separation and affect the distribution of analytes. To date, numerous combinations of QuEChERS salts have been proposed. MgSO_4_, NaCl, Na_2_SO_4_, and NaOAc were the most suitable applicants. The acetate method was performed by applying Na_2_SO_4_, NaCl, and NaOAc as drying salts. Since MgSO_4_ has been shown to promote the chelation of quinolones [21], it was substituted by Na_2_SO_4_, NaCl, and NaOAc.

**Figure 2:**
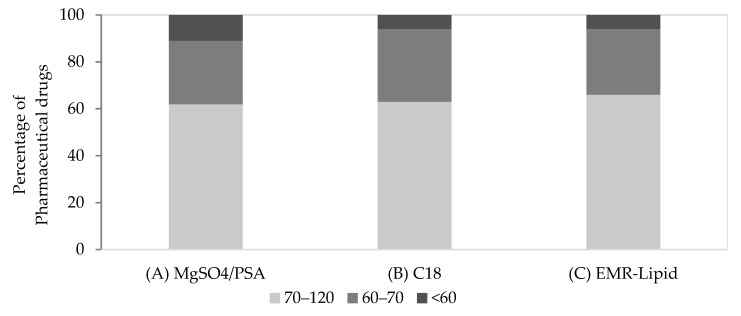
Percentage of pharmaceutical drugs exhibiting recoveries within different ranges (70–120%, 60–70% and <60%) using different sorbents in the clean-up step. (**A**) MgSO_4_/PSA, (**B**) C18, and (**C**) EMR-Lipid in milk.

Secondly, the optimization of the clean-up step of the acetate method was performed. The sorbents assayed were PSA/MgSO_4_, C18, and EMR-Lipid. PSA is a weak anion exchanger sorbent with the ability to remove sugars, organic acids, fatty acids, and polar pigments, while its chemical structure contributes to a high chelating effect [45]. PSA is a sorbent frequently used in the clean-up step of milk extracts for the removal of co-extracted phenolic substances. Anhydrous MgSO_4_ reduces the volume of the aqueous phase by hydration, whereas non-polar interfering substances such as sterols and long-chain aliphatic compounds could be removed by applying C18 sorbent in the purification step [18,32,46]. However, an excessive amount of C18 can also adsorb lipophilic drugs [47]. C18 sorbents have already been reported in the extraction of veterinary drug residues from milk samples, with and without PSA [6,32,48,49]. EMR-Lipid is a newly invented material applied to difficult matrices [23,24,25,26] that offers the promise of highly selective lipid removal, without analyte retention. The following approaches were evaluated for the purification step: (A) 25 mg of PSA and 150 mg of MgSO_4_; (B) 50 mg of C18; and (C) 0.5 g of EMR-Lipid. Figure 2 shows the percentage of target compounds obtained with recoveries <60%, between 60 and 70%, and between 70 and 120%, according to the above combinations. As can be seen, both (B) and (C) clean-up approaches exhibited similar results.

More specifically, in approaches B and C, respectively, 11 and 12 target compounds showed recoveries in the range of 70–120%, and 6 and 5 compounds gave recoveries in the range of 60–70%. In addition, one compound revealed a recovery of <60% in both clean-up approaches. Approach (A) exhibited more target compounds with recoveries below 60%. Thus, approaches (B) and (C) appeared to be more suitable for extract purification. Furthermore, the effectiveness of the purification step was verified by chromatograms with no interfering peaks. Thus, milk samples were further validated according to the previously described and optimized QuEChERS extraction procedure (see Section 3.4.), followed by optimized clean-up approaches (approaches B and C).

### 2.2. Validation of the Proposed Methods

**Figure 3:**
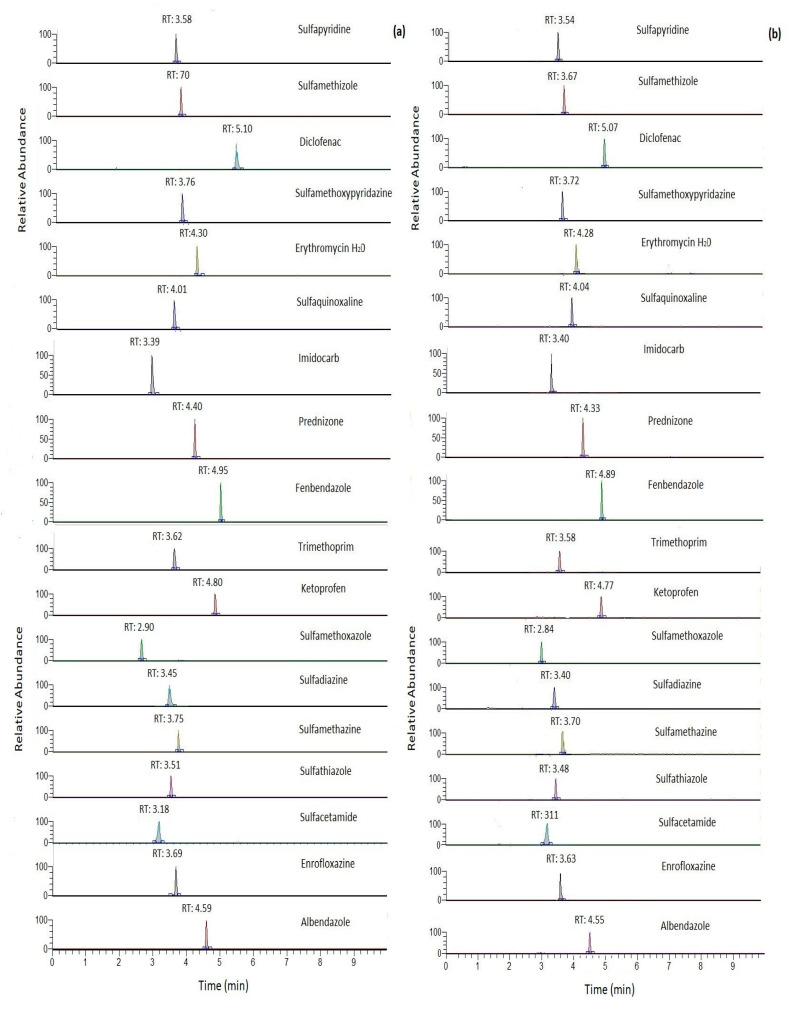
Extracted ion chromatogram (XIC) obtained for target compounds for (**a**) clean-up: C18 (approach B) and (**b**) clean-up EMR-lipid (approach C) at concentration level of 50 μg/kg of milk.

The validation parameters of the modified QuEChERS procedure for milk with two clean-up approaches are shown in Table 1 and Table 2. As we can see in Table 2, the modified “AOAC 2007.01” QuEChERS method with different clean-up steps (approaches B and C) presented excellent linearity in the tested concentration ranges, with correlation coefficient values ≥ 0.99 in all cases. The extracted ion chromatograms (XIC) of target compounds obtained after the two clean-up approaches, (a) C18 and (b) EMR-Lipid, at a concentration of 50 μg/kg in milk samples are shown in Figure 3. Peak areas did not differ greatly between the two approaches with higher values recorded in most cases (11 analytes) using EMR-Lipid.

The trueness and precision of the method were determined in recovery studies with fortified samples at two concentration levels (8 and 50 μg/kg) assayed six times on the same day and six successive days (Table 1). The recoveries at the 8 and 50 μg/kg levels for all the investigated compounds in approach B were within 70–120% with associated RSDs of 19.1%. More specifically, relative recoveries ranged between 75% (sulfathiazole) and 120% (ketoprofen). Intra-day precision in milk ranged from 0.1% for sulfathiazole to 19.0% for fenbendazole, and inter-day precision in milk ranged from 4.3% for diclofenac to 19% for enrofloxacin. In approach C, the recoveries were between 65.1% (diclofenac) and 120.1% (fenbendazole). Intra-day and inter-day precision in milk ranged from 1.9% for albendazole to 9.4% for imidocarb and from 0.4% for enrofloxacin to 17.5% for ketoprofen, respectively.

The LODs and the LOQs of the method are also presented in Table 2. LODs, in approach B, ranged between 0.09 μg/kg for trimethoprim and 15.1 μg/kg for diclofenac, and LOQs ranged between 0.3 μg/kg (trimethoprim) and 50 μg/kg (diclofenac and sulfadiazine), whereas the LODs ranged between 0.09 μg/kg (imidocarb) and 3.1 μg/kg (ketoprofen), and the LOQs were between 0.28 μg/kg (imidocarb) and 10 μg/kg (ketoprofen) in approach C.

**Figure 4:**
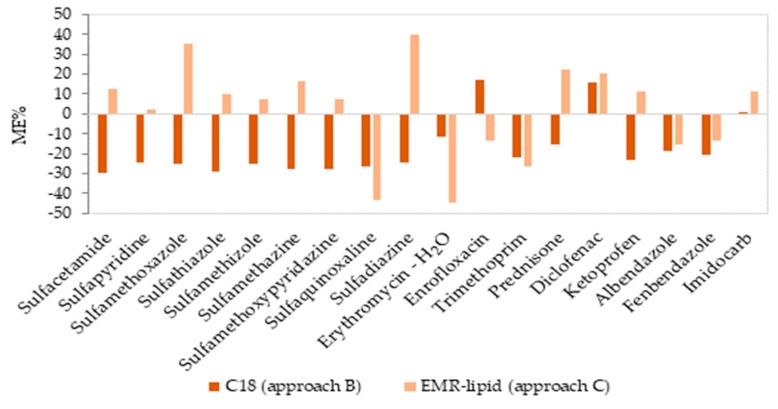
Matrix effect (%ME) values obtained from the slopes of the solvent and matrix-matched calibration curves in milk for two approaches in clean-up step.

The matrix effect values, determined as a signal suppression or enhancement, are illustrated in Figure 4. The ionization efficiency of the analytes may be affected by matrix interferences; thus, calibration curves were established with and without a matrix to evaluate the degree of ion suppression or signal enhancement. In approach B, some of the compounds (six compounds in milk) presented a low matrix effect (between −18.6% and 16.9%), and the rest of the compounds presented a medium matrix effect (twelve compounds between −29.8 to −20.6%). In approach C, twelve compounds presented a low matrix effect, with values between −15.6% and 16.7%, and the rest of the compounds (six compounds) presented a medium matrix effect (between −44.5% and 39.9%).

Similar recoveries were found in both approaches at concentration levels of 8 and 50 μg/kg for all the target compounds. More specifically, relative recoveries were within 75–120% with associated RSDs of 19.1% in approach B and between 65.1% and 120.1% with associated RSDs of 17.5% in approach C. Most of the analytes provided similar LOD and LOQ values in both approaches B and C, except two compounds (sulfadiazine and diclofenac) presented higher values in approach B.

The main features of the developed methods proposed in the current study can be compared with those reported in previously published methods, concerning QuEChERS extraction and LC-MS techniques for the evaluation of pharmaceutical drug residues in fatty matrices. Zhao and Lucas [50] assessed the performance of different sorbents (EMR-Lipid, C18 dSPE, and Z-Sep) for screening veterinary drugs in fatty matrices such as bovine liver. The matrix co-extractive removal efficiency, the accuracy, and the precision of the protocol using the EMR-Lipid sorbent for the clean-up step showed better results compared to the other sorbents for the target compounds. On the other hand, Anumol et al. [24] compared two sample preparation approaches using EMR-Lipid and C18 dSPE for the clean-up step for veterinary drugs on fatty samples, including bovine tissues. The results showed that the use of the EMR-Lipid sorbent gave cleaner extracts and improved results for some less polar compounds, such as anthelmintics and tranquilizers, compared to the usage of C18 sorbent. However, the use of the EMR-Lipid sorbent showed much lower recoveries for β-lactam antibiotics and some polar drugs. Furthermore, Tuzimski and Rejczak [49] evaluated nitroimidazole residues in bovine milk by applying different sorbents (Z-Sep, Z-Sep+, PSA, C18, and EMR-Lipid) for sample purification. Similar recoveries were observed by applying both C18 and EMR-Lipid. The application of C18 sorbent obtained recoveries ranging between 56 and 77%, with standard deviation (SD) < 19%, whereas the use of EMR-Lipid provided recoveries of 48 to 77% with SD < 17%. The utilization of PSA sorbent for the purification step revealed higher recoveries than the other sorbents, ranging from 51 to 85%, with SD < 10% (n = 3) for all analytes; thus, the sorbent PSA was used for the purification step. Moreover, according to Jia et al. [8], different sorbents (including PSA, C18, EMR, and Z-Sep, each of them being tested in combination with MgSO_4_) were evaluated determining veterinary drugs, mycotoxins, and pesticides in bovine milk. The use of both C18 and Z-Sep sorbents to purify the extract performed better than the other two sorbents with more target recoveries falling in the range of 70% to 120%. However, C18 sorbent was preferred for the clean-up step as Z-Sep is not as widely used as C18 in residue analysis. For comparison with low-resolution MS/MS techniques, Jia et al. [8] used QuEChERS extraction with C18 sorbent for the purification step and UHPLC-Qtrap-MS for the determination of multi-class contaminants in milk. This study showed LOD values ranging from 0.01 to 1 µg/kg, and LOQ values were in the range of 0.05 to 5 µg/kg. On the other hand, Castilla-Fernández [25] evaluated two different clean-up steps, (a) EMR-Lipid sorbent and (b) SPE, using UHPLC-QqQ-MS for the analysis of veterinary drugs in milk. The LOQs ranged from 0.01 to 18.25 μg/kg. LC-QqQ-MS and QuEChERS extraction were used for the determination of veterinary drugs in milk by Bang Ye et al. [18]. The method was validated with LOQ values of 5–15 μg/kg. Guo et al. [47] developed a multi-residue method for the determination of 103 veterinary drug residues in milk and dairy products. The method was based on QuEChERS extraction and d-SPE C18/Na_2_SO_4_, combined with an LC-QqQ-MS system, and showed LOQ values in the range of 0.1 to 25 μg/kg for milk. In the current study, the LODs ranged between 0.09 and 15.1 μg/kg, and LOQs were in the range of 0.3 to 50 μg/kg in approach B, whereas the LODs ranged between 0.09 and 3.1 μg/kg, and the LOQs were between 0.28 and 10 μg/kg in approach C. LOQ values fulfilled the MRLs set by the EU for milk in both cases. A comparison of sample preparation methods and analytical techniques for the analysis of pharmaceutical compounds in milk are presented in Table 3.

The LR mass analyzers presented much greater sensitivity (lower LOQs) in most cases than the Orbitrap instrument, while the Orbitrap instrument had better selectivity, measuring accurate mass for both parent and fragmented product ions. The current work offers the comparison of a hybrid QuEChERS–sonication extraction combined with different clean-up steps and UHPLC-HR-Orbitrap-MS capability for the determination of drug residues in milk against previous published methods using mainly LC-LR-MS/MS instrumentation.

**Figure 5:**
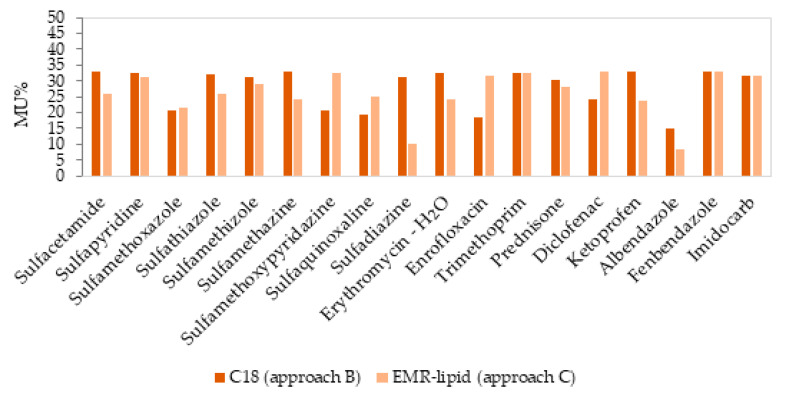
Measurement uncertainty (MU%) for milk in a concentration level of 8 μg/kg (n = 6) for two clean-up approaches.

The expanded MU was estimated individually for each pharmaceutical drug as twice the value of the uncertainty (k = 2, confidence level 95%), and the resulting values are presented in Table 4 and illustrated in Figure 5. In a concentration of 8 μg/kg, the MU values were in the range between 14.80% (albendazole) and 33.04% (fenbendazole and sulfacetamide) in approach B and between 9.94% (sulfadiazine) and 32.78% (diclofenac), in approach C. Both approaches exhibited similar results or better MU values in approach C, except for three compounds (Sulfamethoxypyridazine, diclofenac, and enrofloxacin) that presented higher values, but they were still in compliance with the requirement (50%) of the EU guidance document SANTE/12682/2019 [51].

**Table 3:** Comparison of recent studies regarding extraction and LC-MS techniques for the analysis of pharmaceutical drugs in milk with the current study.

Food Matrix	Compounds	Extraction Method	Clean-Up Method	LC-MS Technique	Acquisition Mode	Linearity	Recovery (%)	LOQs (μg/kg)	Reference
Milk	18 pharmaceutical drugs	Sonication/modified QuEChERS	EMR-Lipid/C18	LC-hybrid LTQ/Orbitrap-MS	Full MS/dd-MS^2^ Resolution 60,000/15,000 FWHM	0.9903	65.1–120.1	0.28–10	Current study
Bovine milk	209 veterinary drugs, mycotoxins, and pesticides	Modified QuEChERS	C18	LC-QTRAP-MS	MRM-IDA-EPI mode	0.99	51.20–129.76	0.05–5	[8]
Milk/dairy products	103 veterinary drugs	Modified QuEChERS	C18 and Na_2_SO_4_	LC-QqQ-MS	MRM mode	0.9902	31.1–120.7	0.5–50	[47]
Goat milk	19 quinolones	“Buffered CEN 15662” QuEChERS	C18 and Na_2_SO_4_	LC–QqQ-MS	MRM mode	0.9853	73.4–114.2	5–15	[18]
Cow milk	66 veterinary drugs	Solvent extraction	EMR-Lipid/SPE	LC-QqQ-MS	MRM mode	0.998	70–120	0.02–18.25	[25]
Milk, cheese, and whey	36 antibiotics	SLE	C18	LC-Q Exactive Orbitrap-MS	Full scan: resolution at 50,000 FWHM	0.995	70–120	1–50 (CC_β_)	[52]

The Horwitz equation for the 8 μg/kg fortification level exhibited an acceptable PRSD_WR_ = 33.1%. Table 4 shows the HorRat value, which was estimated for each one of the pharmaceutical drugs. The HorRat ratio value in all cases was <1, varying between 0.42 (prednisone) and 0.59 (trimethoprim and fenbendazole) in approach B and between 0.42 (erythromycin—H_2_O) and 0.60 (enrofloxacin) in approach C. To conclude, the applied method has better precision than the maximum allowed.

### 2.3. Preliminary Application Study

**Figure 6:**
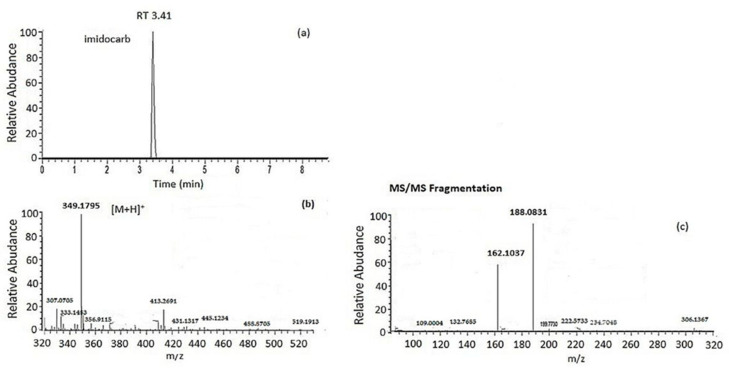
(**a**) Extracted ion chromatogram (XIC), (**b**) full-scan accurate mass parent ion spectrum, and (**c**) MS/MS data obtained for imidocarb in a milk sample.

The optimized method was applied to 10 samples of cow’s milk purchased from Greek markets as a preliminary application case study. The analysis revealed that one of the samples contained a pharmaceutical drug. More specifically, imidocarb was detected only in one sample in the concentration of 18 μg/kg, but the concentration found was far below the MRLs (50 μg/kg) set by the EU for milk (Figure 6). MS^2^ data are depicted in Figure 6c. Two fragment ions were obtained for imidocarb: the first one with an elemental composition of C_12_H_12_O_2_, an *m*/*z* of 188.08313, and a mass error of −0.272 ppm and the second one with an elemental composition of C_11_H_14_O, an *m*/*z* of 162.10373, and a mass error of −1.151 ppm. Imidocarb is an antimicrobial agent that is widely used for the treatment of many diseases in cattle, such as babesiosis and anaplasmosis. Significant residues of imidocarb remain in bovine edible tissues [53] and milk [54] after dosing in cattle. If the recommended withdrawal periods for the drug are not properly implemented, it can lead to the occurrence of imidocarb in edible tissues or milk and eventually in humans through the consumption of contaminated foodstuff.

## 3. Materials and Methods

### 3.1. Chemicals, Reagents, and Samples

Analytical standards (high purity, >98%) of pharmaceutical compounds (sulfacetamide, sulfapyridine, sulfamethoxazole, sulfathiazole, sulfamethizole, sulfamethazine, sulfamethoxypyridazine, sulfaquinoxaline, sulfadiazine, enrofloxacin, trimethoprim, albendazole, erythromycin—H_2_O, prednisone, diclofenac, fenbendazole, and imidocarb) were purchased from Sigma-Aldrich (Darmstadt, Germany), while ketoprofen was acquired from Tokyo Chemical Industry (Oxford, UK). Individual stock solutions of each compound were prepared in methanol and kept in amber glass bottles at −20 °C.

Methanol, acetonitrile, and water (LC–MS grade) were received from Fisher Scientific (Leicestershire, UK). Acetic acid and formic acid (purity, 98–100%) were obtained from Merck KGaA (Darmstadt, Germany). Ultrapure water was produced in the lab by a Milli-Q water purification system (Millipore, Temecula, CA, USA).

The salts/sorbents used in the QuEChERS extraction were purchased as follows: anhydrous magnesium sulfate (MgSO_4_), sodium sulfate (Na_2_SO_4_), C18 (LiChroprep RP-18, 40–64 μm), and trisodium citrate dehydrate (C_6_H_5_Na_3_O_7_ · H_2_O) were purchased from Merck (Darmstadt, Germany); sodium acetate (NaOAc) and sodium chloride (NaCl) were purchased from Riedel-de Haën (Hannover, Germany); primary secondary amine (PSA; 40 μm) and Enhanced Matrix Removal-Lipid (EMR-Lipid) sorbent were purchased from Agilent Technologies (Waldbronn, Germany); sodium citrate dibasic sesquihydrate (C_6_H_6_Na_2_O_7_ · 1.5H_2_O) and ethylenediaminetetraacetic acid tetrasodium salt dihydrate (EDTA) were purchased from Sigma-Aldrich (Steinheim, Germany); and syringe filters (polytetrafluoroethylene, 0.22 μm) were purchased from Millipore (Cork, Ireland). Additionally, 50 mL and 15 mL propylene centrifuge tubes were used.

All milk samples for analysis were of Greek origin. Full-fat (3.5%) milk was used to optimize and validate the analytical method. Cow’s milk samples (a total of 10 samples, all full-fat) were purchased from various local supermarkets and markets in Ioannina, Epirus region, NW Greece. Once transferred to the laboratory for analysis, the samples were kept at 4 °C in amber glass bottles until analysis.

### 3.2. Preliminary Experiments

Initially, three primary QuEChERS methods were investigated. These experiments were aimed to achieve the best extraction efficiency for the target compounds. Ten grams of milk sample were weighed into a 50 mL polypropylene centrifuge tube, spiked at 20 μg/kg fortification level, shaken for 1 min, and extracted as follows below:

(a) Extraction method A (“Original” QuEChERS): Solvent: 10 mL of acetonitrile; extract salts: 4 g of MgSO_4_ and 1 g of NaCl.
(b) Extraction method B (“AOAC 2007.01” QuEChERS) [39]: Solvent: 10 mL of acetonitrile containing 1% acetic acid; extract salts: 6 g of MgSO_4_ and 1.5 g of NaOAc.
(c) Extraction method C (“Buffered CEN 15662” QuEChERS) [40]: Solvent: 10 mL of acetonitrile; extract salts: 4 g of MgSO_4_, 1 g of NaCl, 1 g of C_6_H_5_Na_3_O_7_⋅H_2_O. and 0.5 g of C_6_H_6_Na_2_O_7_⋅1.5H_2_O.

In all cases, after the addition of the salts, the tubes were shaken for 1 min and centrifuged for 5 min at 4000 rpm. Then, 1 mL of the supernatant was transferred into a 15 mL centrifuge tube containing 25 mg of PSA and 150 mg of MgSO_4_ for the clean-up step (clean-up approach A). The tubes were shaken for 1 min and centrifuged for 5 min at 4000 rpm. The supernatant was transferred to a glass testing tube, evaporated to dryness under a gentle stream of nitrogen, and then reconstituted in 1 mL of H_2_O:MeOH (90:10 *v*/*v*) acidified with 0.1% formic acid. The sample was filtered using syringe membrane filters (polytetrafluoroethylene, 0.22 μm) before injection into UHPLC-LTQ/Orbitrap MS.

### 3.3. UHPLC-Orbitrap MS Parameters

The UHPLC system (Accela, Thermo Fisher Scientific, Bremen, Germany) was coupled to a hybrid LTQ Orbitrap XL Fourier transform mass spectrometer (Thermo Fisher Scientific, Bremen, Germany) fitted with an Ion Max electrospray ionization probe. The full scan was applied in positive ionization mode, with a mass range of 120–1000 Da and a mass resolving power of 60,000 FWHM. Extracted ion chromatograms were used for initial identification followed by data-dependent MS/MS (resolution set at 15,000 FWHM), using collision-induced dissociation (CID) with normalized collision energy (NCE) at 35% for all analytes. The mass accuracy tolerance window was set to 5 ppm. Target compounds, retention times, and detection parameters for the data-dependent acquisition (full MS/dd-MS^2^) analysis are presented in Table 5. The main parameters of the mass spectrometer were as follows: spray voltage, 4 kV; tube lens, 90 V; sheath gas flow rate, 35 arbitrary units (au); auxiliary gas flow rate, 10 au, capillary temperature, 320 °C. The scans were applied by targeting the automatic gain control (AGC) at 4 × 10^5^ ions. To process the data. Thermo Xcalibur 2.1 software (Thermo Electron, San Jose, CA, USA) was used.

UHPLC was equipped with an Accela AS autosampler (model 2.1.1) and an Accela quaternary gradient pump. A reversed-phase Fortis C18 (Fortis Technologies, Neston, UK) analytical column (50 × 2.1 mm, 1.7 μm particle size) was used to achieve separation, and column oven temperature was maintained at 35 °C. Mobile phase was a mixture of solvent A, H_2_O acidified with 0.1% formic acid, and solvent B, MeOH acidified with 0.1% formic acid. The elution gradient started at 95% A; remained at 95% for 1 min; changed to 30% after 1 more min; changed to 0% after 3 more min, where it remained for 2 min; and finally returned to the initial conditions. The total run time was 10 min. The injection volume was 5 μL, and the flow rate was 250 μL/min.

### 3.4. QuEChERS Extraction Procedure

The sample extraction and clean-up procedures followed in method B (“AOAC 2007.01” QuEChERS) [39] were selected as the most efficient method, and some further modifications have been studied for optimizing the method. Ten grams of the milk sample were weighed into a 50 mL polypropylene centrifuge tube and spiked at 8 and 50 μg/kg fortification levels. A total of 10 mL of EDTA 0.1 M was added, and the tubes were immediately shaken for 1 min. Then, 10 mL of acetonitrile containing 3.35% formic acid were added, and the tubes were shaken again for 1 min. The mixture was placed in a sonication bath (37 kHz, 100 W, Elmasonic P, Singen, Germany) for 20 min. Afterwards, 4 g of Na_2_SO_4_, 1.2 of NaCl, and 0.7 g of NaOAc were added; shaken again for 1 min; and centrifuged for 5 min at 4000 rpm. Two alternative clean-up approaches were evaluated: 50 mg of C18 (clean-up approach B) and 0.5 g of EMR-Lipid (clean-up approach C). A total of 1 mL of the organic upper phase was transferred into a 15 mL polypropylene centrifuge tube containing 50 mg of C18. On the other hand, 5 mL of the organic upper phase was transferred into a 15 mL polypropylene centrifuge tube containing 0.5 g of EMR-Lipid, which was firstly conditioned with 2.5 mL of Milli-Q water and shaken for 30 s. Then, it was transferred into a 15 mL polypropylene centrifuge tube containing 1.6 g of MgSO_4_ and 0.4 g of NaCl. For both the above clean-up approaches, after the addition of the sorbents, the tubes were vigorously shaken for 1 min and then centrifuged for 5 min at 4000 rpm. Finally, 1 mL of the supernatant was transferred to a glass testing tube, evaporated to dryness under a gentle stream of nitrogen at 40 °C, and reconstituted into 1 mL of H_2_O:MeOH (90:10, *v*/*v*) acidified with 0.1% formic acid. Finally, the sample was filtered using syringe membrane filters (polytetrafluoroethylene, 0.22 μm) before being injected into the UHPLC-LTQ/Orbitrap MS instrument.

### 3.5. Method Validation

Matrix-matched standard calibration curves prepared in H_2_O:MeOH (90:10, *v*/*v*) acidified with 0.1% formic acid were used for the quantification of drug residues. Blank milk samples were used for the construction of matrix-matched calibration curves. The method was validated by determining analytical parameters such as sensitivity/linearity, recovery, precision, limit of detection (LOD) and limit of quantification (LOQ), matrix effects (ME), and measurement uncertainty (MU), following the European Commission (EC) documents 2002/657/EC and N° SANTE/12682/2019 [51,55].

The linearity of the method was checked in the range of 5 to 100 µg/kg, using weighted least-squares regression and expressed as a determination coefficient (R^2^). Blank samples of biologically produced milk (previously analyzed for drug residues) were spiked with a pharmaceutical drug mixture at 8 and 50 μg/kg fortification levels to determine recovery rates. In all cases, six replicates (n = 6) were prepared for each spike level. The method’s trueness and precision were studied via the mean recoveries (Rec%) and the relative standard deviation (RSD%) at 8 and 50 μg/kg. Repeatability (%RSD_r_) and intermediate precision (%RSD_WR_) were tested on the same day (n = 6) and for six consecutive days, respectively. According to SANTE guidance documents, mean recoveries should range between 70 and 120%, with an associated RSD ≤ 20%, for all analytes. In some cases, mean recovery rates can be accepted outside the range of 70–120% if RSD ≤ 20%, but the mean recovery must not be lower than 30% or higher than 140%. The sensitivity of the method was evaluated by determining limits of quantification (LOQs) and limits of detection (LODs) of the target compounds using a signal-to-noise ratio (S/N) of 3 and 10, respectively. According to N° SANTE/12682/2019, LOQ values should be ≤ MRLs. The matrix effect (ME) values for the target compounds were studied by comparing a calibration curve prepared in milk extract and a calibration curve prepared in the solvent, at the same concentration range, according to the formula below:

(1) $$\%ME=\frac{slope\;of\;calibration\;curve\;in\;matrix}{slope\;of\;calibration\;curve\;in\;solvent}-1\times 100$$

*%ME* is a useful parameter to assess the effectiveness of the method since co-extractants could increase or reduce the analytical signal. *%ME* values <±20%, <±20–±50%, and >±50% are considered low, medium, and high, respectively [56].

Six replicate analyses performed on different days at a level of 8 μg/kg were used to evaluate the expanded MU for the two clean-up approaches. A default expanded MU of 50% should not be overstepped (equivalent to a confidence level of 95% and a coverage factor of 2). Furthermore, another practice for evaluating acceptable measurement precision is to use the Horwitz equation and the Horwitz ratio (HorRat) [57].

## 4. Conclusions

Different approaches have been effectively applied in the extraction and clean-up steps for the analysis of pharmaceutical drugs in milk using modified QuEChERS extraction combined with sonication and UHPLC-Orbitrap MS. The acidified acetate method was selected as more efficient, and the optimization of the purification revealed C18 and EMR-Lipid as the best dispersive SPE (dSPE) agents. The optimized methods were further validated and the parameters of linearity, trueness, precision, LOD, and LOQ and expanded uncertainty fulfilled the requirements according to the SANTE guidelines, while the HorRat values revealed a better method precision than allowed. The EMR-Lipid approach provides better validation parameter values for most of the analytes. The method was applied to real samples, revealing the existence of one pharmaceutical drug (imidocarb) in one milk sample. The developed method can serve as an effective and easy approach for the determination of a wide range of pharmaceutical drug residues in milk.

## Figures and Tables

**Table 1:** Comparison of the methods applying two different clean-up approaches, C18 (approach B) and EMR-Lipid (approach C): intra- and inter-day mean relative recoveries (Rec%), repeatability (expressed as relative standard deviation (%RSD_r_) and intermediate precision (expressed as relative standard deviation (%RSD_WR_), at two different spiked levels (n = 6).

Pharmaceutical Drug	Clean-Up: C18 (Approach B)								Clean-Up: EMR-Lipid (Approach C)							
	Intra-Day				Inter-Day				Intra-Day				Inter-Day			
	8 μg/kg		50 μg/kg		8 μg/kg		50 μg/kg		8 μg/kg		50 μg/kg		8 μg/kg		50 μg/kg	
	Rec%	RSD_r_%	Rec%	RSD_r_%	Rec%	RSD_WR_%	Rec%	RSD_WR_%	Rec%	RSD_r_%	Rec%	RSD_r_%	Rec%	RSD_WR_%	Rec%	RSD_WR_%
Sulfacetamide	81.3	12.9	66.6	16.5	89.0	11.6	93.0	9.2	80.5	6.8	81.8	3.7	87.1	4.1	94.1	5.7
Sulfapyridine	81.8	6.1	92.6	13.0	90.3	13.3	94.6	8.0	80.0	4.1	82.1	2.5	83.1	17.1	85.4	15.6
Sulfamethoxazole	102.1	9.2	105.0	12.5	100.3	15.5	101.3	11.0	78.4	6.2	80.5	3.1	89.9	3.0	100.2	13.6
Sulfathiazole	75.0	0.1	79.3	12.2	82.3	9.1	86.0	9.3	82.5	6.9	85.9	4.8	88.0	4.2	90.3	8.4
Sulfamethizole	85.8	6.1	99.0	13.5	83.3	15.4	93.0	7.8	78.3	3.6	80.0	2.2	80.4	9.3	82.4	13.0
Sulfamethazine	85.8	6.1	103.3	11.3	80.3	17.6	92.6	10.0	74.5	4.3	80.2	5.2	97.1	10.6	98.6	11.3
Sulfamethoxypyridazine	87.8	15.3	94.6	9.3	93.1	11.0	94.0	9.6	71.5	5.3	75.0	4.1	83.7	9.8	88.6	14.3
Sulfaquinoxaline	94.0	6.9	88.3	12.9	82.0	18.6	90.3	8.5	70.6	4.0	80.0	2.2	88.7	4.3	92.2	9.5
Sulfadiazine	85.8	6.1	103	13.6	81.3	12.2	85.3	9.8	76.0	3.6	80.0	2.1	98.9	4.4	100.5	7.3
Enrofloxacin	92.0	0.2	95.6	12.7	110.5	19.0	112.6	15.2	81.9	4.6	89.4	7.5	84.0	0.4	86.0	12.6
Trimethoprim	83.6	8.0	96.6	7.7	83.3	15.4	89.0	8.2	81.0	3.1	83.1	3.8	97.9	13.0	99.4	9.7
Erythromycin—H_2_O	92.0	6.6	94.3	7.5	107.3	11.9	111.0	14.8	88.7	6.5	92.0	4.8	110.4	6.0	110.6	9.2
Prednisone	85.6	10.9	95.3	11.7	95.3	14.0	97.6	15.8	83.9	4.9	89.2	7.3	88.0	5.6	91.8	8.5
Diclofenac	83.6	8.9	87.6	18.7	83.1	19.0	87.5	4.3	65.1	3.2	68.5	5.8	75.8	2.17	76.0	8.2
Ketoprofen	118	9.4	120.0	10.9	112	14.1	112.2	9.5	101.3	7.7	103.9	5.3	101.4	17.5	108.2	15.1
Albendazole	98.1	11.8	99.3	14.9	91.5	15.1	93.3	10.6	78.8	1.9	82.3	4.4	99.1	3.8	101.2	13.8
Fenbendazole	75.1	10.5	77.3	19.0	110.5	11.1	113.3	10.1	84.4	7.8	91.0	5.1	113.3	9.9	120.1	9.2
Imidocarb	117.0	5.2	118.1	7.8	99.3	9.6	100.6	16.7	81.5	4.1	85.6	9.4	85.7	5.5	93.0	14.7

**Table 2:** Comparison of the methods applying two different clean-up approaches, C18 (approach B) and EMR-Lipid (approach C): linearity, limits of detection (LODs), limits of quantification (LOQs), as well as maximum residue levels (MRLs) for pharmaceutical drug residues in milk.

Pharmaceutical Drug	C18 (Approach B)			EMR-Lipid (Approach C)			
	Linearity (R^2^)	LOD (μg/kg)	LOQ (μg/kg)	Linearity (R^2^)	LOD (μg/kg)	LOQ (μg/kg)	MRL (μg/kg)
Sulfacetamide	0.9936	2.80	9.25	0.9980	1.67	5.08	100
Sulfapyridine	0.9929	0.92	2.77	0.9974	0.61	1.87	100
Sulfamethoxazole	0.9987	0.64	1.92	0.9945	0.73	2.24	100
Sulfathiazole	0.9900	1.89	6.25	0.9973	2.42	7.33	100
Sulfamethizole	0.9983	3.33	10.0	0.9963	2.38	7.20	100
Sulfamethazine	0.9987	0.92	2.77	0.9939	2.87	8.68	100
Sulfamethoxypyridazine	0.9944	0.98	2.94	0.9934	1.10	3.35	100
Sulfaquinoxaline	1.0000	0.72	2.17	0.9999	1.54	4.67	100
Sulfadiazine	0.9983	15.1	50.0	0.9984	2.97	9.01	100
Enrofloxacin	0.9953	0.69	2.08	0.9915	0.10	0.30	100
Trimethoprim	0.9904	0.09	0.30	0.9981	1.03	3.11	100
Erythromycin—H_2_O	0.9962	0.37	1.13	0.9952	2.38	7.22	50
Prednisone	0.9994	1.26	3.80	0.9983	0.41	1.26	150
Diclofenac	0.9930	15.1	50.0	0.9973	2.49	7.53	100
Ketoprofen	0.9998	0.53	1.78	0.9949	3.10	10.0	50
Albendazole	0.9989	0.14	0.44	0.9961	1.88	5.71	100
Fenbendazole	0.9920	0.66	2.00	0.9955	1.04	3.16	10
Imidocarb	0.9969	0.83	2.50	0.9903	0.09	0.28	50

**Table 4:** Measurement uncertainty MU (%) calculated for milk at a concentration level of 8 μg/kg (k = 2, confidence level 95%) and HorRat values for different clean-up approaches.

Pharmaceutical Drug	C18 (Approach B)		EMR-Lipid (Approach C)	
	MU%	HorRat	MU%	HorRat
Sulfacetamide	33.04	0.57	25.89	0.48
Sulfapyridine	32.37	0.58	31.13	0.57
Sulfamethoxazole	20.54	0.48	21.44	0.56
Sulfathiazole	32.16	0.58	26.12	0.57
Sulfamethizole	31.23	0.58	29.01	0.58
Sulfamethazine	32.99	0.57	24.27	0.52
Sulfamethoxypyridazine	20.85	0.53	32.49	0.57
Sulfaquinoxaline	19.29	0.56	24.88	0.56
Sulfadiazine	31.23	0.58	9.94	0.51
Erythromycin—H_2_O	32.62	0.58	23.96	0.42
Enrofloxacin	18.30	0.55	31.75	0.60
Trimethoprim	32.62	0.59	32.62	0.59
Prednisone	30.30	0.42	28.26	0.57
Diclofenac	23.97	0.55	32.78	0.59
Ketoprofen	32.96	0.43	23.56	0.46
Albendazole	14.80	0.45	8.55	0.50
Fenbendazole	33.04	0.59	32.76	0.44
Imidocarb	31.55	0.43	31.80	0.58

**Table 5:** UHPLC–LTQ Orbitrap MS analysis data. Target pharmaceutical drugs, retention times (t_R_), and detection parameters for full MS/dd-MS^2^ analysis.

Pharmaceutical Drug	t_R_ (min)	Pseudo-Molecular Ion [M + H]^+^	Theoretical Mass (*m*/*z*)	Experimental Mass (*m*/*z*)	Ring Double Bond Equivalent (RDBE)	Mass Accuracy	Fragment Ions 35% NCE
Sulfacetamide	3.11	C_8_H_11_N_2_O_3_S	215.0484	215.0485	4.5	−0.416	108.0488/156.0112
Sulfadiazine	3.4	C_10_H_11_N_4_O_2_S_2_	251.0597	251.0614	7.5	−0.091	156.0124/158.0027
Sulfamethazine	3.7	C_12_H_15_N_4_O_2_S	279.091	279.0928	7.5	−0.082	108.0448/204.0450
Sulfamethizole	3.67	C_9_H_11_N_4_O_2_S_2_	271.0318	271.0335	6.5	0.024	156.0123/177.9751
Sulfamethoxazole	2.84	C_10_H_12_N_3_O_3_S	254.0594	254.0609	6.5	0.045	160.0878/195.0923
Sulfamethoxypyridazine	3.72	C_11_H_13_N_4_O_3_S	281.0703	281.0722	7.5	0.045	126.0669/156.0125
Sulfapyridine	3.54	C_11_H_12_N_3_O_2_S	250.0645	250.0661	7.5	0.105	156.0106/184.0861
Sulfaquinoxaline	4.04	C_14_H_13_N_4_O_2_S	301.0754	301.0772	10.5	0.090	146.0721/156.0123
Sulfathiazole	3.48	C_9_H_10_N_3_O_2_S_2_	256.0209	256.0227	6.5	0.021	97.7709/156.0112
Enrofloxacin	3.63	C_19_H_23_FN_3_O_3_	360.1718	360.172	9.5	0.010	316.1840/360.1739
Trimethoprim	3.58	C_14_H_19_N_4_O_3_	291.1462	291.147	7.5	3.548	123.0657/261.0792
Erythromycin—H_2_O	4.28	C_37_H_66_NO_13_	716.458	716.4581	5.5	0.066	126.1283/389.2128
Prednisone	4.33	C_21_H_27_O_5_	359.1853	359.1879	8.5	−0.001	147.0822/341.1767
Diclofenac	5.07	C_14_H_12_Cl_2_NO_2_	296.0239	296.024	9	−0.002	250.0151/214.0338
Ketoprofen	4.77	C_16_H_15_O_3_	255.1016	255.1018	9.5	0.114	194.004
Albendazole	4.55	C_12_H_16_N_3_O_2_S	266.0958	266.0976	6.5	0.098	191.0138/234.0712
Fenbendazole	4.89	C_15_H_14_N_3_O_2_S	300.0801	300.0823	10.5	−0.08	268.0558
Imidocarb	3.40	C_19_H_21_N_6_O	349.1771	349.1796	12.5	−0.103	162.1036/188.0830

## Data Availability

Data are included in the manuscript.
